# Segmental concatenation of individual signatures and context cues in banded mongoose (Mungos mungo) close calls

**DOI:** 10.1186/1741-7007-10-97

**Published:** 2012-12-03

**Authors:** David AWAM Jansen, Michael A Cant, Marta B Manser

**Affiliations:** 1 Animal Behaviour, Institute of Evolutionary Biology and Environmental Studies, University of Zurich, Winterthurerstrasse 190, 8057 Zurich, Switzerland; 2 Daphne du Maurier, Centre for Ecology and Conservation, College of Life and Environmental Sciences, University of Exeter, Cornwall Campus, TR10 9EZ, UK

**Keywords:** vocal signature, vocal cue, syllable, close call, segregation of information, graded calls, banded mongoose, segmental concatenation

## Abstract

**Background:**

All animals are anatomically constrained in the number of discrete call types they can produce. Recent studies suggest that by combining existing calls into meaningful sequences, animals can increase the information content of their vocal repertoire despite these constraints. Additionally, signalers can use vocal signatures or cues correlated to other individual traits or contexts to increase the information encoded in their vocalizations. However, encoding multiple vocal signatures or cues using the same components of vocalizations usually reduces the signals' reliability. Segregation of information could effectively circumvent this trade-off. In this study we investigate how banded mongooses (*Mungos mungo*) encode multiple vocal signatures or cues in their frequently emitted graded single syllable close calls.

**Results:**

The data for this study were collected on a wild, but habituated, population of banded mongooses. Using behavioral observations and acoustical analysis we found that close calls contain two acoustically different segments. The first being stable and individually distinct, and the second being graded and correlating with the current behavior of the individual, whether it is digging, searching or moving. This provides evidence of Marler's hypothesis on temporal segregation of information within a single syllable call type. Additionally, our work represents an example of an identity cue integrated as a discrete segment within a single call that is independent from context. This likely functions to avoid ambiguity between individuals or receivers having to keep track of several context-specific identity cues.

**Conclusions:**

Our study provides the first evidence of segmental concatenation of information within a single syllable in non-human vocalizations. By reviewing descriptions of call structures in the literature, we suggest a general application of this mechanism. Our study indicates that temporal segregation and segmental concatenation of vocal signatures or cues is likely a common, but so far neglected, dimension of information coding in animal vocal communication. We argue that temporal segregation of vocal signatures and cues evolves in species where communication of multiple unambiguous signals is crucial, but is limited by the number of call types produced.

## Background

Nonhuman-animals (hereafter referred to as animals) have finite vocal repertoires and are anatomically constrained in the number of different call types they can produce [1,2]. These constraints limit the variation of a species' vocal repertoire and may have played an important role in the evolution of meaningful combinations of calls [3,4]. Another possible way to encode senders' related information in vocalizations is through vocal signatures (specifically for individual identity and/or group membership) and/or cues (related to all other individual traits or context; hereafter we refer to both signatures and cues as vocal cues) [5-8].

Although individual identity is the most commonly reported vocal cue [8], animal vocalizations have also been shown to contain cues for group identity [8-12], size [13-15], male quality, [14,16,17], sex [18,19], and reproductive state [20]. Animals can encode vocal cue information using two general sets of acoustic properties. Firstly, spectral features, such as fundamental frequency or harmonic-to-noise ratio, can differ between individuals to encode for instance individuality [8]. Additionally, a number of recent studies have shown that filter-related formants are a reliable indication of body size and male quality [13-15,21]. The importance of these formants has mainly been shown in larger mammals, such as rhesus macaques (*Macaca mulatta*) [13], dogs (*Canis familiaris*), red deer (*Cervus elaphus*) [14,22] or fallow deer (*Dama dama*) [15]. Secondly, vocal cue information can be encoded in vocalizations through temporal features. Individual cues encoded by variance in the temporal features, such as duration or temporal arrangement of frequency elements have been reported for species such as the big brown bat (*Eptesicus fuscus*), pallid bat (*Antrozous pallidus*), and cricket species (*Gryllidae spp*.) [8]. All of these vocal cues potentially provide useful information to the receiver whenever variation between categories is larger than the within-category variation.

Many animal calls contain combinations of multiple different vocal cue types [5-8]. The expression of these multiple vocal cues typically correlates with different frequency-related acoustic parameters. The individualistic grunts of baboons (*Papio spp*.) are, for instance, audibly distinct in different behavioral contexts [23-25]. However, acoustic space is limited and many acoustic parameters are correlated with one another. Therefore, the amount of frequency related variation that can be used by signalers to encode different vocal cues is ultimately constrained. This constraint can result in a trade-off between the various kinds of information and typically reduces reliability of at least one of the vocal cues [26,27]. For instance, the use by signalers of available variation for individual recognition conflicts with the need for stereotypic characteristics for group recognition in bird song [26]. Briefer *et al*. [27] showed a similar trade-off between the vocal cues for identity (stable over time) and male quality (variable over time) in fallow deer. Segregation of information could partially resolve this trade-off by expressing functionally different cues in temporally distinct call segments or in different acoustic features [26,27]. In the white-crowned sparrow (*Zonotrichia leucophrys pugetensis*), for example, individual identity and group membership are segregated into the distinct note complex and trill phrases of its song respectively, thus avoiding a trade-off in reliability between the vocal cues [28]. Similar segregation of information (though not specifically referred to) has been shown in the songs of meadow pipits (*Anthus pratensis*) [29], rock hyraxes (*Procavia capensis*) [30], humpback whales (*Megaptera novaeangliae*) [31] and killer whales (*Orcinus orca*) [32]. Although this principle was proposed by Marler in 1960 [26], currently no studies have shown temporal segregation in the form of segmental concatenation within a single syllable call type. Such within-syllable encoding would have analogues with 'phonological' or segmental concatenation used in human language [33].

Contact calls are among the most common vocalizations produced by both mammalian and bird species. In a variety of species, contact calls seem to function to coordinate movements and cohesion of individuals on a range of spatial scales, concurrently with various behaviors and in a variety of social systems [34,35]. Contact calls have been shown to contain individual vocal cues [8,12,36] and group membership vocal cues [9,11,12,37]. Contact calls can also contain multiple vocal cues as has been shown in baboons [23-25] and meerkats (*Suricata suricatta*) [12]. In some species contact calls seem to function predominantly over mid- to long-distance, while in others contact calls play a more important role in short-distance communication. It has been suggested that these short distance close calls, often low in amplitude and pitch and consisting of a single syllable, are better described as close calls [12,38]. Such close calls have the potential to provide constant information about the individual characteristics of the signaler and are likely used to monitor changes in behavior and relative spatial positioning of members in social groups [12,34,35,39,40].

Cooperatively breeding banded mongooses (*Mungos mungo*) are small (≤ 2 kg) social carnivores that show high group cohesion. They live in mixed sex groups, with an average of around 20 individuals, but groups occasionally grow to more than 70 individuals [41]. They forage together as cohesive units and cooperate in pup care, predator avoidance and territory defense [41-43]. During foraging, banded mongooses move in and out of dense vegetation with many position shifts, both in distance to nearest neighbor and in relative position within the group. They regularly dig for food items in the soil with their heads down. Besides digging they also search for food on the surface, but this is mainly done in the thickets (see Table 1 for details). They are often visually constrained during foraging and, therefore vocalizations play a critical role in keeping individuals informed of changes in the social and ecological environment. Banded mongoose use a range of graded vocalizations to coordinate behaviors and to maintain group cohesion [44,45]. One of the most commonly emitted call types is the close call and previous work has demonstrated the presence of an individual vocal cue within the call [46]. Subsequent field observations suggested additional graded variation in the close calls, which appeared to be related to the behavioral context experienced by the signaler (personal observations DJ). We, therefore, investigated whether banded mongooses' close calls contain multiple vocal cues and how these vocal cues are encoded in the temporal and frequency related aspects of this graded single syllable call type.

**Table 1:** Definitions of the different behavioral context used for the acoustical analysis.

Context	Definition
Digging	The signaler was digging for or eating food, and the animal was not moving and its head was facing downward.
Searching	The signaler was searching for food in and around the same foraging patch, with head predominately facing downward.
Moving	The signaler was moving between foraging patches but within the spatial cohesion of the group and with head predominately facing forward.

## Results

**Figure 1:**
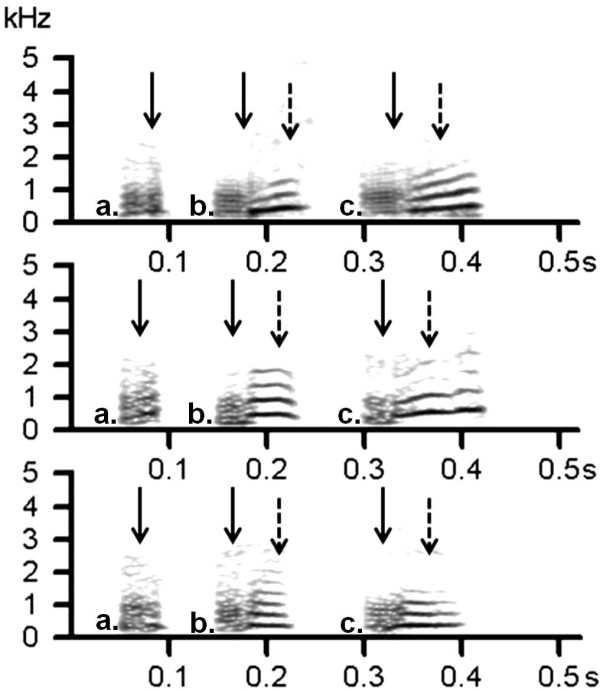
**Spectrograms of banded mongoose close calls**. Spectrograms of close calls of the three individuals (in rows 1 to 3) associated with the three different behavioral contexts: **a**.) digging; **b**.) searching; **c**.) moving between foraging patches. The calls in the first and second row are of females, while calls in the third row are of a male. Calls of the individuals in the second and third row are from the same social pack. The solid black arrows indicate the individually stable foundation of the call, while the dashed arrows indicate the harmonic tonal segment (Hamming, FTT = 1024, overlap = 97.87%, frequency resolution = 43 h).

The acoustic structure of close calls in banded mongoose varied significantly between individuals and behavioral contexts. Specifically, the initial noisy segment of the call remained stable within an individual in all of the quantified behavioral contexts, while a gradation was detected in the subsequent harmonic tonal segment (Figure 1, Additional files 1, 2, 3). Close calls could be individually distinguished statistically in all four groups (total number of individuals = 36, range per group 7 to 14). Correct cross validation probabilities varied between 40% and 61% for the initial noisy segment and the whole call, and bootstrapping showed that all classification probabilities were much higher than that expected by chance (Table 2). The cross-validation probabilities for the harmonic part of the call were considerably lower at 11% to 25% and were not significantly different than expected by chance (Table 2). A group-specific vocal cue was found in the noisy segment of the call (number of correctly cross-classified elements (*ncce*) = 44.47, *P *= 0.038, *n *= 36), but not for the whole call (*ncce *= 38.08, *P *= 0.27), nor for the harmonic segment (*ncce *= 44.47, *P *= 0.038, *n *= 36). No evidence for a sex-specific vocal cue was found in either the whole call (*ncce *= 60.35, *P *= 0.54, *n *= 36), or the initial noisy part (*ncce *= 64.23, *P *= 0.19, *n *= 36).

 A cross-classified permutated discriminant function analysis (pDFA) showed that, overall, close calls were correctly classified to the appropriate behavioral context (Table 1) based on their acoustic structure (*ncce *= 44.22, *P <*0.001, *n *= 20). Specifically, the harmonic extension of the close calls varied significantly and was correctly classified according to the behavioral context (*ncce *= 78.04, *P *= 0.009, *n *= 18), whereas the initial noisy segment of the call was not (*ncce *= 19.87, *P *= 0.79, *n *= 20). Thereby, the harmonic segment was either not present or of a very short duration in the digging context (*mean *± *sd*; 0.01 *± *0.02 s), while its duration increased in the searching context (0.05 *± *0.03 s). The longest and most pronounced harmonic segments were observed in the moving context (0.08 *± *0.03 s). For pairwise comparisons of the acoustic structures between behavioral contexts, see Table 3.

**Table 2:** Individual vocal cue classification

Group	#*^a^*	Random (%)*^b^*	Whole call CV-values (%)	Noisy segment CV-values (%)	Harmonic segment CV-values (%)
1B	8	12.5	48.1***	45.0***	25.0
1H	14	7	26.1*	40.0***	11.4
11	7	14	42.0***	48.0***	22.0
15	7	14	61.5***	61.1***	22.5

The percentage of correct classification after cross-validation (CV) to individuals within each of the four study groups compared to that expected by chance; Results for the whole call, noisy segment and harmonic segment are given; *p*-values are derived from bootstrapping method [46]; *^• ^p *≤ 0.1, * *p *≤ 0.05, ** *p *≤ 0.01, *** *p *≤ 0.001

*^a ^*Number of individuals tested

*^b ^*Expected by chance.

**Table 3:** Behavioral vocal cue classification

Part analyzed	Behavior	Individuals	ncce
Whole call	digging-searching	30	3.340^•^
	digging-moving	25	40.640***
	searching-moving	20	30.610***

Noisy segment	digging-searching	30	1.500
	digging-moving	25	34.850
	searching-moving	20	23.100

Harmonic segment	digging-searching	18	78.040***
	digging-moving	30	77.440***
	searching-moving	30	67.600**

The pDFA classification results for pairwise comparisons between behaviors.; Results for the whole call, noisy segment and harmonic segment are given.; The results of the pDFA is the number of correctly cross-classified elements (ncce); *^• ^p *≤ 0.1, * *p *≤ 0.05, ** *p *≤ 0.01, *** *p *≤ 0.001

The calls used to generate the results of this article are available in the Labarchives repository http://dx.doi.org/10.6070/H4W37T8Q[47].

## Discussion

Banded mongoose close calls, consisting of a single syllable, were not only individually distinct, but also differed in their acoustic structure depending on the current behavior of the signaler. This acoustic variation depended on the behavioral context encoded within a harmonic extension of the basic noisy segment of the close call. To our knowledge this is the first example of temporal segmentation as a means of encoding multiple types of information within a call consisting of a single syllable in an animal vocalisation. Variation in spectral aspects (for example, fundamental frequency) of the more noisy call element verify previous findings of individual cues in close calls of banded mongoose [46]. In that study, Müller and Manser [46] showed, using playback experiments that pups are able to discriminate between close calls of their escorting adult and the close calls of other adults. Their results suggest that individual vocal cues of these close calls are meaningful to receivers. Additionally, here we found group specific vocal cues. Such cues of group identity may arise because the physical characteristics that determine vocal characteristics of an individual (for example, vocal fold length (for F0) and/or vocal tract length (for formants)) are, on average, more similar among group members than non-group members. Another possibility in species with vocal flexibility and where individuals change groups is that individuals converge to match the vocal group cue of the new group after switching [48,49]. At present it is unknown which of these two processes is applicable for the banded mongoose. In contrast, temporal features (for example, duration) of the tonal harmonic segment of the call seem to encode the behavioral vocal cues. Future research using playback experiments will need to be conducted to investigate if behavioral context vocal cues are used by receivers.

While many animal signaling systems, including human speech, use concatenation of acoustically-separate syllables to enrich and extend the signaling space (for example, birdsong [28,29], rock hyraxes (*Procavia capensis*) [30] or cetacean species [31,32]), human speech also encodes information into individual syllables. By combining stop consonants with different vowels at a phonological level, syllables are created that have different meanings. Thus, a stop consonant like/b/versus/p/can be combined with a vowel like/a/or/o/to create a richer signaling unit than either class (that is, stop consonants or vowel) alone could provide. Such combinations (versus 'syntactic' concatenation of syllables and words) are a core feature of the phonological component of human spoken language [33]. The temporally segmented fashion in which banded mongooses encode multiple cues into a single syllable close call is analogous to this system. Moreover, our study provides an example of a discrete individual 'element' in a graded call containing information regarding individuality. The noisy, yet stable, segment of the close call, explained almost as much individual variation as the whole call. This implies that, despite the graded nature of the close call, individual identity is encoded in a discrete way.

The functional aspect of the discrete identity cue in combination with a graded behavioral cue seems analogous to human communicative contexts, when sender and receiver cannot see each other. For example, in the drum or whistle languages of tribes in the remote and isolated conditions of mountainous or densely forested areas, discrete signals are used to announce identity and other information to avoid ambiguity [50,51]. Similarly, in radio conversations in aviation between pilots and control towers, identity and additional information are shared in a highly standardized order (that is, You Me Where What With; chapter 5, in [52]). Signals in these 'conversations' are intentionally chosen for their clarity to the receivers [53,54]. In particular in species that are constantly moving as a cohesive unit, in their search for food or shelter, and where the identification of an individual cannot be based on its spatial position, acoustic individual identity may be a crucial aspect for the successful operation of the system. This is true for banded mongooses where coordination of foraging and movement facilitates the successful functioning of the overall social system. Temporal segregation of vocal cues may enable banded mongooses to reliably encode dual information sets regarding an individual's identity and its current behavioral context. Our study on banded mongoose close calls demonstrates temporal segregation within a single syllable call type. However, reviewing spectrograms of other species' calls, available in the literature, reveal that our findings may not be unique to banded mongooses. For example, the well-known 'whine-chuck' advertisement call of the túngara frog (*Physalaemus pustulosus*) provides another example of segregation of information within a single syllable, where whines encode the species identity and the chucks refer to male quality [55,56]. Such a system is highly advantageous in providing detailed reliable information in an otherwise ambiguous graded system. Human speech [6,54,57,58], and elements of some other species' vocal repertoires such as Barbary macaque (*Macaca sylvanus*) [59,60], chimpanzee (*Pan troglodytes*) [61,62] and Japanese macaque (*Macaca fuscata*) [54] are, from the production side, classified as a graded system, yet perceived by the receivers as discrete [6,59-61,63]. Graded signals have the potential to convey subtle and complex information, but potentially suffer from heightened ambiguity [54,64]. This ambiguity can partly be resolved by meaningful, within-category, classification of a graded signal into perceptually discrete signals [64,65]. It has been hypothesized that this perception of a graded continuum as a series of discrete units was a crucial stage in the evolution of human language [63,64]. This analogous ability in banded mongoose demonstrates that animal communication systems also have the potential to convey a rich set of information in an acoustically sophisticated way.

Recent studies have shown that some free ranging primates use meaningful call- and element-combinations to vastly increase the range of information that can be decoded by listeners [3,4,66-71]. This may be particularly important for forest species living in dense vegetation, where no visual cues can be used to verify the information content or context of the signal [3,4]. In the same way, we suggest that species that use vocal cues ultimately benefit from an increased informational repertoire and, therefore, similar species demonstrating combinatorial calling behavior could be expected to make use of multiple vocal cues and benefit from temporal segregation of information. Vocal cues predominantly encode individual related cues of the sender (for example, identity or male quality) and we, therefore, predict temporal segregation to evolve when signalers could benefit from unambiguous multiple vocal cues. Call combinations have been hypothesized to occur in response to discrete external events (for example, alarm calls) or behavioral contexts, but not directly related to characteristics of the signaler [3,71]. Species with graded vocal systems would especially benefit from the use of unambiguous vocal cues, since these would; *i*) avoid the lack of clarity that generally occurs in graded vocalizations, and *ii*) potentially enhance the reliability of categorization by receivers of graded signals into discrete units.

## Conclusion

Our results show that considerable acoustic variation underlies the close calls of banded mongooses with specific information in temporarily segregated vocal cues. Through the segregation of acoustic information, the potential trade-off in reliability between vocal cues can be avoided. Many nonhuman-animals have small vocal repertoires [3,4,72] and call combinations are one way animals can get around the limited information content of a finite vocal repertoire. Here we demonstrate that temporarily distinct acoustic segments relating to specific vocal cues provide an equally effective and reliable solution to this problem and represent an additional dimension to the complexity underlying information coding in animal vocal communication. To what extent these are used throughout the animal kingdom is an important question to be addressed in the future, as it may help us to identify the selective pressures that gave rise to these kinds of abilities in non-human animals and potentially also in humans.

## Methods

### Study population

The study site was located in Uganda, in the Queen Elizabeth National park (0°12S; 29°54E). The study site and the habituated population have been described in detail elsewhere [41,73]. During the period of data collection (February 2009 to July 2011), the study population consisted of six habituated groups and three semi-habituated groups, with group sizes ranging from 6 to 50+ individuals. In five groups, most individuals were habituated to a level that allowed us to follow them with a microphone and to do detailed focal watches. As part of the Banded Mongoose Research Project long-term data collection protocol, all animals were tagged with subcutaneous transponders (TAG-P-122GL, Wyre Micro Design Ltd., UK), whereas for field identification individuals were given small hair cuts or, for less habituated fully grown adults, color-coded plastic collar (weight ≤ 1.5 g, regularly checked to ensure a loose fit) [73].

### Recording methods

All close calls used in the acoustic analysis were recorded from well-habituated adult (≤ 1 year) banded mongooses at a distance of approximately 1 to 2 m, using a Sennheiser directional microphone (ME66/K6 and a MZW66 pro windscreen, frequency response 40-20000 Hz ± 2.5 dB, Old Lyme, Connecticut, U.S.A.) connected to a Marantz PMD-660 solid state (Marantz Japan Inc.) or a M-Audio Microtrack II (Avid Technology USA Inc.). Calls were recorded in wav format with 16 bits and 44.1 kHz sample rate. Calls were recorded as part of detailed behavioral focal watches or during *ad libitum *sampling recording sessions. In 2009, audio recordings were made at the same time as video focal watches to record behavior (Canon HF100); in 2010/11, commentaries on behavior were added to the audio recording. It was noted whether the individual was a.) digging, b.) searching, or c.) moving within the foraging patch of the group (Table 1 and for details of behavior see [74]). For the acoustic analysis, calls with high signal-to-noise ratio were selected, using Avisoft SASLab Pro 5.18 (R. Specht, Berlin, Germany) [75]. Only individuals for which we had at least five calls in at least two of the behavioral contexts were included in the analysis. For individuals where more than five calls were available, we randomly selected five calls [76]. The calls are available in the Labarchives repository http://dx.doi.org/10.6070/H4W37T8Q[47].

### Acoustic analysis

A 1,024-point fast Fourier transformation (Hamming window; time step: 0.07 ms; overlap: 96.87%; frequency range: 44.1 kHz; frequency resolution: 43 Hz) was conducted for all calls, using Avisoft. We manually assigned labels to the whole call, the noisy base of the call and, if present, the harmonic part of the call (Figure 1). We then used a batch processing option to obtain automatic measurements for 12 parameters (Table 4). The minimum frequency is the lowest frequency of the amplitude exceeding this threshold (-20 dB), while the maximum frequency is the highest frequency of the amplitude exceeding this threshold. The bandwidth is the difference between minimum and maximum frequency. These quartile variables characterize the distribution of energy across the spectrum and indicate the frequency below which 25, 50 or 75%, respectively, of the energy can be found. The distance between quartile 75% and quartile 25% is a measure of the pureness of the sound. The 50% quartile also indicates the mean frequency. All mean frequency measures were obtained from the mean spectrum of each call or call component, while the three quartiles were also measured from the point within the call or call component that had the maximum amplitude [75]. We also calculated the transition onset (fundamental frequency (F0) at the onset of call minus F0 at the middle of the call) and offset (F0 at the middle of the call minus F0 at the end of the call) [12]. The automatic measurements were checked by visual inspection of the graphic results of the measurements in the spectrograms.

**Table 4:** Overview of parameters used and their values per call segment (mean+(sd))

Acoustic parameters		Digging	Whole call Moving	Searching
Duration	(s)	0.05 ± (0.02)	0.12 ± (0.04)	0.09 ± (0.04)
Bandwidth	(mean Hz)	1,472 ± (428)	1,526 ± (378)	1,439 ± (382)
F0	(mean Hz)	263 ± (100)	467 ± (89)	380 ± (110)
Onset		20 ± (150)	-456 ± (1,752)	-205 ± (1,020)
Offset		-133 ± (814)	204 ± (1,694)	-184 ± (1,781)
Max freq.	(Hz)	1,587 ± (427)	1,675 ± (373)	1,575 ± (375)
Min. freq.	(Hz)	114 ± (31)	149 ± (55)	135 ± (43)
Peak frequency	(mean Hz)	370 ± (167)	490 ± (123)	404 ± (106)
Quartile 25%	(mean Hz)	430 ± (74)	525 ± (82)	469 ± (73)
Quartile 50%	(mean Hz)	753 ± (96)	918 ± (213)	846 ± (199)
Quartile 75%	(mean Hz)	1,426 ± (539)	2,730 ± (1,748)	2,217 ± (1,615)
Quartile 25%	(max Hz)	454 ± (77)	533 ± (77)	481 ± (78)
Quartile 50%	(max Hz)	802 ± (123)	942 ± (184)	898 ± (240)
Quartile 75%	(max Hz)	1,803 ± (1,033)	2,734 ± (1,745)	2,507 ± (1,738)

		Digging	Initial noisy segment Moving	Searching

Duration	(s)	0.04 ± (0.01)	0.03 ± (0.01)	0.03 ± (0.01)
Bandwidth	(mean Hz)	1,534 ± (457)	1,542 ± (473)	1,534 ± (426)
F0	(mean Hz)	225 ± (94)	249 ± (138)	218 ± (92)
Onset		45 ± (138)	127 ± (963)	44 ± (186)
Offset		-46 ± (129)	-146 ± (951)	-79 ± (713)
Max freq.	(Hz)	1,646 ± (455)	1,654 ± (470)	1,650 ± (417)
Min. freq.	(Hz)	112 ± (26)	112 ± (28)	116 ± (30)
Peak frequency	(mean Hz)	380 ± (186)	378 ± (195)	363 ± (178)
Quartile 25%	(mean Hz)	439 ± (74)	473 ± (90)	450 ± (79)
Quartile 50%	(mean Hz)	754 ± (92)	838 ± (163)	795 ± (110)
Quartile 75%	(mean Hz)	1,329 ± (387)	2,300 ± (1,744	1,787 ± (1,250
Quartile 25%	(max Hz)	465 ± (77)	497 ± (96)	473 ± (83)
Quartile 50%	(max Hz)	797.3 ± (100)	914 ± (270)	849 ± (157)
Quartile 75%	(max Hz)	1,654 ± (865)	2,847 ± (1,997)	2,234 ± (1,612)

		Digging	Harmonic segment Moving	Searching

Duration	(s)	0.03 ± (0.02)	0.08 ± (0.04)	0.06 ± (0.03)
Bandwidth	(mean Hz)	1,185 ± (405)	1,307 ± (394)	1,283 ± (474)
F0	(mean Hz)	350 ± (70)	472 ± (83)	410 ± (82)
Onset		-10 ± (59)	-83.0 ± (1,444)	-6 ± (789)
Offset		-177 ± (1,294)	-19 ± (1,134)	-176.4 ± (1,284)
Max freq.	(Hz)	1,343 ± (408)	1,572 ± (390)	1,513 ± (463)
Min. freq.	(Hz)	158 ± (83)	264 ± (93)	230 ± (97)
Peak frequency	(mean Hz)	350 ± (88)	485 ± (115)	409 ± (84)
Quartile 25%	(mean Hz)	414 ± (76)	536 ± (89)	471 ± (81)
Quartile 50%	(mean Hz)	769 ± (316)	967 ± (285)	895 ± (301)
Quartile 75%	(mean Hz)	2,346 ± (1,514)	2,861 ± (1,927)	2,784 ± (2,037)
Quartile 25%	(max Hz)	419 ± (76)	546 ± (91)	491 ± (98)
Quartile 50%	(max Hz)	802 ± (280)	976 ± (210)	940 ± (317)
Quartile 75%	(max Hz)	2,505 ± (1,598)	2,797.6 ± (1,821)	2,801 ± (1,956)

### Statistical analysis

We conducted all analyses in R, version 2.14 (R Development Core Team 2010), using the software packages 'car' [77], 'kla' [78], 'lme4' [79], and 'MASS' [80]. The analyses described below were done on the whole call, on the 'noisy' segment of the call, and if present, on the 'harmonic segment' of the call (Figure 1). We performed linear mixed effect models (lmer) on the acoustic variables to calculating variance inflation factors and obtaining a subset of acoustic parameters that was free from multicollinearity as this is essential for the proper functioning of the discriminant function analysis (DFA). It has been argued that conventional DFA provides grossly inflated levels of overall significance of discriminability when using multiple samples of the same individual [76] and that in such cases a permuted discriminant function analysis (pDFA) should be used. We controlled for repeated sampling of groups and individuals by fitting 'individual' nested in 'group' as a random factor [81]. We used an adapted form of the variance inflation factors (VIF) analysis that worked directly on predictors in lmer models (Austin Frank, pers. comm.) to detect multicollinearity in the acoustic parameters. Only parameters with a VIF ≤ 2.5 were included in the analyses. The remaining parameters were entered into a DFA to determine the correct classification probabilities of close calls to i.) behavior while controlling for individual and ii.) individuals while controlling for behavior. DFA identifies linear combinations of predictor variables that best characterize the differences among groups and combines the variables into one or more discriminant functions, depending on the number of groups to be classified [78,80]. This method of analyses provides a classification procedure that assigns each call to its appropriate class (correct assignment) or to another class (incorrect assignment). A stepwise variable selection was performed for the DFA. The initial model consisted of the parameters that remained after the selection with the linear effect model and the VIF analysis; in subsequent steps new models were generated by either including or excluding single variables in the model. This resulted in a performance measure for these models that were estimated by cross-validation, and if the maximum value of the chosen criterion was better than the previous model, the corresponding variable was included or excluded. This procedure was stopped once the new best value, after including or excluding any variable, did not exceed a 5% improvement. The number and type of variables included in the analysis differed per analysis and sub-analysis. Duration was included in all behavioral context specific tests. The number of variables included was smaller than the number of individuals included in the test [76]. For external validation, we used a leave-one-out cross-validation procedure and estimated the significance levels for correct statistical assignment of calls using *post hoc *'bootstrapping' analyses. This method determined the probability that a cross-validated correct assignment value was achieved by chance [46]. Our data for behavioral, group, and sex vocal cues were two factorial (test factor and individual) and contained five call examples per individual, we, therefore, used a crossed pDFA (Mundry, pers. comm.). Furthermore, to ensure no differences resulted from variation in sex or group, we also performed pDFAs while keeping these two additional variables constant. We performed four pDFAs to test for overall and the pairwise comparison between behavioral contexts. In addition, we performed two additional pDFAs to test for the group cue and sex cues (both while controlling for individual). From one of the groups, we did not have calls from a large enough number of individuals to perform a classification analysis, and, therefore, the group vocal cue analysis was conducted on four groups only.

### Ethical note

This research was carried out under license from the Uganda National Council for Science and Technology, and all procedures were approved by the Uganda Wildlife Authority. Trapping and marking procedures, which are part of the long-term research program, followed the guidelines of the Association for the Study of Animal Behavior [43,73].

## Abbreviations

DFA: discriminant function analysis; F0: fundamental frequency; lmer: linear mixed effect models; ncce: number of correctly cross-classified elements; pDFA: permutated discriminant function analysis; VIF: variance inflation factors.

## Competing interests

The authors declare that they have no competing interests.

## Authors' contributions

DJ designed the study, collected data in the field, analyzed the data and wrote up of the paper. MC helped to write the paper and provided logistical support in the field. MM designed research and helped to write the paper. All authors read and approved the final manuscript.

## Supplementary Material

Additional file 1 **Banded mongoose 'digging' close call Example of a digging close call**. Click here for file

Additional file 2 **Banded mongoose 'searching' close call Example of a searching close call**. Click here for file

Additional file 3 **Banded mongoose 'moving' close call Example of a moving close call**. Click here for file
